# Systematic screening of isogenic cancer cells identifies DUSP6 as context-specific synthetic lethal target in melanoma

**DOI:** 10.18632/oncotarget.15863

**Published:** 2017-03-02

**Authors:** Stephanie Wittig-Blaich, Rainer Wittig, Steffen Schmidt, Stefan Lyer, Melanie Bewerunge-Hudler, Sabine Gronert-Sum, Olga Strobel-Freidekind, Carolin Müller, Markus List, Aleksandra Jaskot, Helle Christiansen, Mathias Hafner, Dirk Schadendorf, Ines Block, Jan Mollenhauer

**Affiliations:** ^1^ Department of Molecular Genome Analysis, German Cancer Research Center (DKFZ), 69118 Heidelberg, Germany; ^2^ Institute for Comparative Molecular Endocrinology, Ulm University, 89081 Ulm, Germany; ^3^ Institute for Laser Technologies in Medicine and Metrology, Ulm University, 89081 Ulm, Germany; ^4^ Lundbeckfonden Center of Excellence NanoCAN, Institute of Molecular Medicine, University of Southern Denmark, 5000 Odense, Denmark; ^5^ Molecular Oncology, Institute of Molecular Medicine, University of Southern Denmark, 5000 Odense, Denmark; ^6^ Department of Otorhinolaryngology, Section for Experimental Oncology and Nanomedicine (SEON), University Hospital Erlangen, 91054 Erlangen, Germany; ^7^ Genomics and Proteomics Core Facility, German Cancer Research Center (DKFZ), 69118 Heidelberg, Germany; ^8^ Department of Biotechnology, Mannheim University of Applied Sciences, 68163 Mannheim, Germany; ^9^ Department of Dermatology, University Hospital Duisburg-Essen, 45147 Essen, Germany and German Cancer Consortium, 69118 Heidelberg, Germany; ^10^ Department of Clinical Genetics, Odense University Hospital, 5000 Odense, Denmark; ^11^ Lundbeckfonden Center of Excellence NanoCAN, Institute of Molecular Medicine, University of Southern Denmark, 5000 Odense, Denmark; ^12^ Molecular Oncology, Institute of Molecular Medicine, University of Southern Denmark, 5000 Odense, Denmark

**Keywords:** cancer, isogenic cell line libraries, functional genomics, melanoma, synthetic lethal

## Abstract

Next-generation sequencing has dramatically increased genome-wide profiling options and conceptually initiates the possibility for personalized cancer therapy. State-of-the-art sequencing studies yield large candidate gene sets comprising dozens or hundreds of mutated genes. However, few technologies are available for the systematic downstream evaluation of these results to identify novel starting points of future cancer therapies.

We improved and extended a site-specific recombination-based system for systematic analysis of the individual functions of a large number of candidate genes. This was facilitated by a novel system for the construction of isogenic constitutive and inducible gain- and loss-of-function cell lines. Additionally, we demonstrate the construction of isogenic cell lines with combinations of the traits for advanced functional *in vitro* analyses. In a proof-of-concept experiment, a library of 108 isogenic melanoma cell lines was constructed and 8 genes were identified that significantly reduced viability in a discovery screen and in an independent validation screen. Here, we demonstrate the broad applicability of this recombination-based method and we proved its potential to identify new drug targets via the identification of the tumor suppressor DUSP6 as potential synthetic lethal target in melanoma cell lines with BRAF V600E mutations and high DUSP6 expression.

## INTRODUCTION

Since its advent, genome-wide mRNA expression profiling has been extensively used to compare tumors versus normal tissues, different stages of cancer progression, expression patterns associated with drug resistance and differences between cancer subtypes. In recent years, next-generation sequencing (NGS) techniques have further enabled researchers to sequence DNA and mRNA for mutations on genome-wide scale. Commonly, these approaches discover several hundreds of genes with recurrent mutations.

The first consensus mutational map generated using NGS identified 189 genes with potentially relevant mutations in driver genes of breast and colon cancer, most of which have an unknown role in cancer [1, 2]. Shah *et al*. recently identified more than 2,000 somatic single nucleotide variations including 107 insertion-deletion mutations in triple negative breast cancer [3]. In addition, four studies identified a cumulated set of 137 genes with potential driver mutations in melanoma [4–7]. Elucidating these genes` functions and interplay is a critical step towards the identification of novel starting points for therapy as, for example, demonstrated by the development of PARP-inhibitors for BRCA1/2-deficient tumors [8].

To translate the results of mutation screens into clinical applications, in-depth characterization of the phenotypic effect of each mutation is essential. However, systematic downstream functional analysis is rarely considered and evaluation commonly limited to a single or few genes [1–7]. The lack of comprehensive functional approaches may partially be due to limitations of existing strategies. Conceptually, lentiviral systems may enable functional studies of large gene sets as they enable efficient stable genomic insertion even in non-dividing cells [9]. A major drawback of this method is that multiple insertions may occur in a cell, leading to unaccounted copy-number variation effects. Moreover, the random genomic integration may create a highly heterogeneous population.

Alternatives that overcome these limitations of lentiviral systems are genome-editing techniques based on, for example, zinc finger nucleases, CRISPR/Cas9 or Transcription Activator-Like Effector Nucleases (TALENs). They enable targeted insertion of genetic changes with high precision at single nucleotide-level. In contrast to lentiviral systems, genome editing allows for the creation of isogenic cells which differ only in the introduced genetic alteration. However, the efficacy of these techniques is variable and often in the lower percent range [10–14].

Site-specific recombination-based systems, such as the Flp-FRT [15] and the Cre-lox system [16], facilitate stable insertion of a recombinase recognition sequence (FRT- or lox-site) into the host cell genome. These sequences can consecutively be used to insert genes into the prepared chromosomal site, allowing for the derivation of isogenic cell lines with high efficacy.

Due to the high degree of standardization, we expected this technique to be suitable for serially analyzing large gene sets. First, we optimized an existing site-specific recombination-based system for the rapid and effective construction of isogenic cell line panels. Second, we extended this system to simplify cell line construction with either constitutive or inducible expression of a single gene or two genes of interest. Subsequently, we demonstrated the successful recombination at a single genomic location in a number of cell lines of different cancer types. We then selected one of these cell lines, A375, as a representative for a proof-of-concept screen, in which we constructed more than a hundred isogenic melanoma cell lines. An independent repetition of the cell line construction procedure was conducted for a subset of these genes to demonstrate the reproducibility of the results. Finally, the top five growth-suppressing genes were further characterized by cell cycle analysis and by generating cell line recombinants for cell lines of other cancer types. A main finding of additional proof-of-concept experiments was that the tumor suppressor *DUSP6* could serve as a potential synthetic lethal drug target in melanoma with BRAF V600E mutations.

## RESULTS

### Re-design of site-specific recombination systems

Site-specific recombination has been used for various applications, such as, an endogenous sequence of the host cell genome used for the insertion of sequences of interest (e.g. the *rosa* locus in mice) [17]. Moreover, exogenous sequences like FRT- or lox-sites can be stably integrated for subsequent gene insertion [15–17]. Remarkably, these techniques have been used extensively for generating transgenic mice, but have not been exploited for *in vitro* functional genomics approaches.

**Figure 1:**
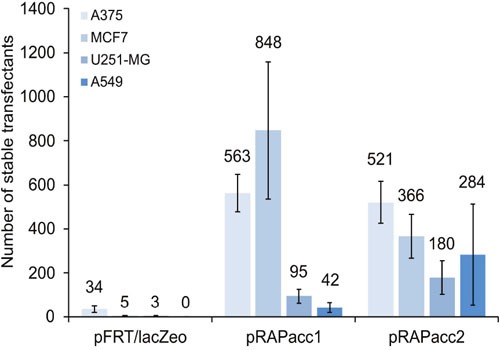
Vector performance in generating FRT-site modified acceptor cell lines Four cancer cell lines were transfected under identical conditions with either pFRT/lacZeo from the commercially available Flp-In system or with pRAPacc1 or 2. After two weeks of selection, the number of lacZ-positive (pFRT/lacZeo) and of EGFP-positive cells (pRAPacc1/2) was scored. Values are averages of at least two independent experiments. Error bars represent standard error of the mean (SEM).

We initially used the commercially available Flp-In system (Life Technologies). The system provides a plasmid (pFRT/lacZeo) to first insert the FRT recombination site into the host cell genome. The FRT-site locates between a cytomegalovirus (CMV) promoter with an ATG start codon that drives expression of a *lacZ*-zeocin resistance fusion gene. Our goal was to insert an expression plasmid hosting a gene of interest as well as a hygromycin resistance gene using the Flp recombinase. The inserted genes would be under the control of the CMV promoter and start codon, thereby eliminating the expression of the zeocin resistance fusion gene. Accordingly, the absence of lacZ positive and zeocin resistant cells, and the presence of hygromycin resistance would indicate successful transfections. As no correspondingly modified cancer cell lines are available, we transfected the frequently used cancer cell lines A375 (melanoma), MCF7 (breast cancer), U251-MG (brain cancer), and A549 (lung cancer). Unfortunately, repeated transfections with the pFRT/lacZeo plasmid yielded low numbers of lacZ-positive cells in three of the four cell lines (Figure 1). Moreover, attempts to generate stable isogenic cell lines via site-specific recombination did not result in any hygromycin-resistant cells (data not shown).

**Figure 2:**
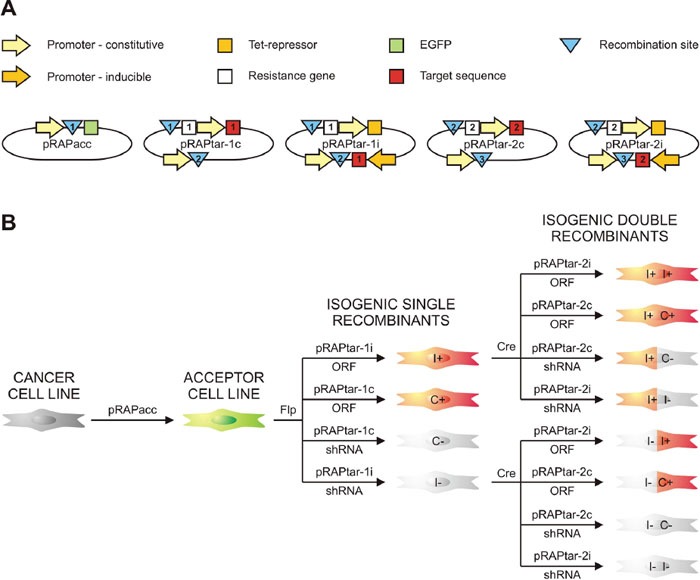
Schematic survey of the system **(A)** Display of the vectors with their most relevant elements. Recombination sites are indicated by blue triangles (1: FRT-site; 2: lox-sites; 3: phiC31 attP site for later expansion). White squares depict resistance genes for selection of isogenic single recombinant cells (ISRs; 1: hygromycin resistance gene) and isogenic double recombinant cells (IDRs; 2: puromycin resistance gene). Red squares symbolize the inserted target sequence, which could be either a gene or a shRNA-coding cassette for knockdown (1: target sequence for first insertion via Flp-FRT; 2: target sequence for second insertion via Cre-lox). **(B)** Schematic survey of part of the permutations of traits that can be generated. Recombination-mediated insertion of a pRAPtar-1 vector in the ACL genome yields selectable ISRs that lack green fluorescence and display either dox-inducible (I) or constitutive (C) expression of the target sequence. ISRs with overexpression of an open reading frame (ORF) or short hairpin RNA-mediated silencing of a gene (shRNA) would show gain (“+”) or loss of function (“-“). Consecutive Cre-mediated insertion of a pRAPtar-2 vector yields selectable IDRs with the desired combination of traits as exemplified for using the two inducible single recombinants.

We therefore created new vectors in which the ATG-FRT cassette was placed upstream of an EGFP reporter using pEGFP-N1 (Clontech) as the backbone (Figure 2A, Supplementary Figure 1 and Supplementary Table 1). This configuration substituted the *lacZ*-zeocin resistance fusion gene with EGFP and a separate, i.e. non-fused, neomycin resistance gene. One vector variant (pRAPacc1) was equipped with the CMV promoter, while the other (pRAPacc2) contained the elongation factor 1-alpha (E1Fα) promoter. Using these newly generated vectors, reporter-positive cells were generated with 15- to 188-fold increased efficacy compared to the original system and, in addition, stably transfected clones of A549 cells were obtained which was not achieved with the pFRT/lacZeo system (Figure 1).

We further modified the expression plasmids (referred to as pRAPtar vectors) to enable serial construction of cell lines with constitutive and inducible expression of single or multiple genes. The pRAPtar-1c was designed for constitutive expression of target genes, while pRAPtar-1i contained a doxycycline (dox)-inducible promoter and a Tet-repressor in *cis* to provide isogenic cells with inducible expression in a single recombination step (Figure 2A and 2B, Supplementary Figure 1). The latter is particularly useful when studying cancer-related genes, since their constitutive expression often causes increased cell death.

In addition to the recombination-activated hygromycin resistance gene, we inserted a lox71-site [16] preceded by an SV40 promoter and an ATG start codon. As a result, the introduction of the first plasmid established a secondary recombination site suitable for the insertion of a plasmid via Cre-lox-mediated recombination. Consequently, a constitutive (pRAPtar-2c) and an inducible version (pRAPtar-2i) of the corresponding expression vector were generated. These carried a lox66-site preceding a start-codon-deficient puromycin resistance gene (Figure 2A, Supplementary Figure 1). Thus, successful Cre-mediated recombination yielded puromycin-selectable cells. Finally, the pRAPtar-2 vectors further contain a third recombination site (phiC31 attP site) [18] to theoretically allow for the consecutive insertion of another sequence (Figure 2B, Supplementary Figure 1).

Accordingly, following stable insertion of the pRAPacc vectors to provide a so-called acceptor cell line (ACL), the system allows for constructing isogenic single recombinant cells (ISRs) via Flp-recombinase that have constitutive or inducible expression of a target sequence. Depending on whether a gene or a knockdown construct (e.g. shRNA or pri-miRNA) is inserted, this results in a cell line for subsequent gain- or loss-of-function studies (Figure 2B). Consecutively, ISRs can be used to insert a second sequence expressed in a constitutive or inducible fashion to obtain isogenic double recombinant cells (IDRs) with combinatorial traits (Figure 2B).

### Construction of acceptor cell lines

We next constructed a panel of ACLs from 10 commonly used cell lines of various cancer types (Table 1). After transfection of pRAPacc vectors and selection of neomycin-resistant clones, we processed 16-54 stable EGFP-positive clones per cell line to identify cell lines suitable as ACLs for further studies.

**Table 1:** Generation of acceptor cell lines

Cell line	Type	Stable clones	Single integration	Rec.+*^a^*	Expr.+*^b^*	ACL Name
Mewo	Melanoma	42	13	4	0	-
SkMel28	Melanoma	20	4	3	0	-
A375	Melanoma	32	7	4	3	N103*^c^*, N104*^c^*, N120*^c^*
H1299	Lung cancer	48	6	6	1	B5*^c^*
A549	Lung cancer	53	12	12	1	X12*^d^*
MCF-7	Breast cancer	42	6	2	1	N107*^c^*
MDA-MB-231	Breast cancer	25	8	8	0	-
U87-MG	Brain cancer	42	4	4	0	-
U138-MG	Brain cancer	54	3	3	0	-
U251-MG	Brain cancer	16	7	4	2	L106*^c^*, L1*^e^*
**Total**	**10**	**374**	**70/374**	**50/70**	**8/50**	**5/10 cell lines**
**Success rate**			**19%**	**71%**	**16%**	**50%**

*^a^* capable of accepting pRAPtar-1 vector by site-specific recombination as judged by obtaining hygromycin-selectable cells lacking green fluorescence; *^b^* capable of expressing target genes in an inducible fashion as judged by fluorescence microscopy and flow cytometry analysis with the HcRed reporter gene in pRAPtar-1i after dox induction; *^c^* generated with pRAPacc2; *^d^* generated with earlier variant of pRAPacc1 (pRAPacc1a) containing an additional TetR, which was non-functional after stable insertion; *^e^* generated with pRAPacc1.

We expected that some clones would host multiple insertions of vectors into the cell's genome, compromising subsequent selection of positive recombinants and conceivably favoring undesired intra- or inter-chromosomal recombination events. Thus, we tested an initial set of 374 individual clones for single integration via Southern blotting, yielding 70 clones (19%), for which single integration could be confirmed (Table 1, Supplementary Figure 2).

Next, the ability to accept an expression plasmid via Flp-mediated recombination was assessed, using the red fluorescent HcRed reporter gene cloned into pRAPtar-1c. Most, but not all, of the clones (50 out of 70; 71%) readily delivered stable hygromycin-resistant ISRs, in which EGFP was switched off and the HcRed gene was constitutively switched on (Table 1), as was determined by fluorescence microscopy.

**Figure 3:**
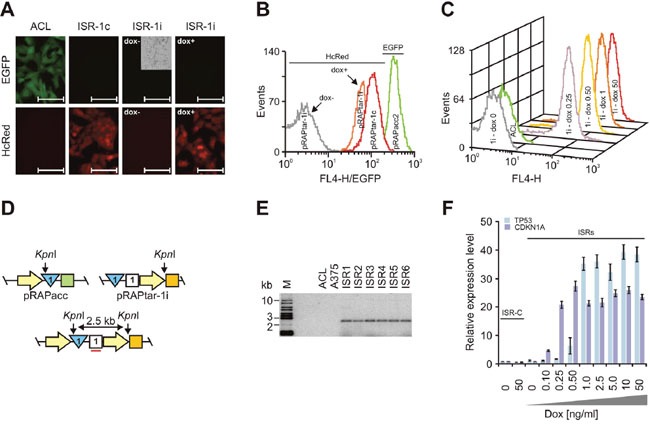
Construction of isogenic single recombinants Analyses used A375-ACLN103 cells. **(A)** Fluorescence microscopy of acceptor cell line A375-ACLN103 (ACL) and isogenic single recombinants with insertion by recombination of constitutively (ISR-1c) and dox-inducibly (ISR-1i) expressed HcRed. The phase contrast inlay (cropped) is to demonstrate the presence of cells. For ISR-1i images were taken 48 h after induction without (dox-) or with (dox+) 50 ng/ml doxycycline. Scale bars: 100 μm. **(B)** Flow cytometry analyses of HcRed expression for the corresponding ISRs and of EGFP expression for A375-ACLN103. **(C)** Flow cytometry analyses of HcRed expression in response to different dox concentrations depicted in ng/ml at the respective curve. The HcRed-negative ACL is included as negative control. **(D)** Configuration of pRAPacc inserted into the host cell genome (top left), part of pRAPtar-1i carrying the *TP53* open reading frame (top right) and after recombination of pRAPtar-1i into the FRT-site in the host cell genome (bottom). Yellow arrow: promoter; blue triangle: FRT-site; green square: EGFP; white square: hygromycin resistance gene; orange square: Tet-repressor. Correct insertion into the genome-localized FRT-site would combine two *Kpn*I restriction sites in such way that a new and unique 2.5-kb restriction fragment emerges that can be detected with a radioactively labeled probe against the hygromycin resistance gene (indicated by the red line). **(E)** Southern blot analysis of *Kpn*I-digested genomic DNA from six independent ISRs with insertion of *TP53* cloned in pRAPtar-1i (ISR1-6), the A375-ACLN103 acceptor cell line used (ACL) and the original non-modified A375 cell line, probing the hygromycin resistance gene. M: size marker. **(F)** *TP53* and *CDKN1A* were used to construct ISRs with pRAPtar-1i. Isogenic control recombinants (ISR-C) with insertion of the empty (i.e. ORF-deficient) pRAPtar-1i vector served as negative controls. Levels of mRNA expression were analyzed 48 h after induction with different dox concentrations by qRT-PCR. Error bars represent SEM.

We further analyzed the performance of the dox-inducible expression vector. Consequently, we used the HcRed gene that was inserted in pRAPtar-1i via Flp-recombinase for testing. Silencing and induction of red fluorescence was detected via microscopy and flow cytometry. The majority of the clones (42 out of 50; 84%) either displayed lack of suppression in the absence of dox or lack of induction after dox addition (summarized in Table 1). The remaining eight ACLs covered five different cancer cell lines (Table 1, Figure 3A and 3B, Supplementary Figure 3A). For a single ACL-pRAPtar-1i clone per cancer cell line we further confirmed inducible HcRed expression quantitatively in response to increasing amounts of dox (Figure 3C, Supplementary Figure 3B). In conclusion, from 374 clones eight ACLs were isolated for the subsequent construction of clones with functional constitutive and inducible gene expression. For five out of 10 cancer cell lines, ACLs could be obtained that passed the described quality filters, corresponding to a 50% success rate.

### Construction of isogenic single and double recombinant cells

To further test properties of the ACLs, we focused on A375-ACLN103 (melanoma) cells. Fluorescence microscopy and flow cytometry confirmed high and homogeneous expression after insertion of the HcRed gene via pRAPtar-1c recombination and quantitatively inducible expression after insertion of the HcRed gene via pRAPtar-1i recombination (Figure 3A-3C). We next inserted the *TP53* gene cloned in pRAPtar-1i into the ACL genome and isolated six independent EGFP-negative clones (*TP53*-ISRs).

Southern blot analyses confirmed *TP53* insertion exclusively into the genomic location flagged by the FRT-site (Figure 3D and 3E). We also inserted *CDKN1A* in pRAPtar-1i and tested inducible expression of the gene alongside *TP53* expression in one of the *TP53*-ISRs. Both clones displayed tunable mRNA expression levels in response to different dox concentrations and showed highly comparable induction kinetics (Figure 3F). In conclusion, the data indicated that ISRs demonstrated uniform induced expression as expected from isogenic cells.

Finally, we tested the construction of inducible double recombinants (IDRs) suitable to express combinatorial traits via Cre-mediated recombination. To facilitate this we used the A375-ACLN103 ISRs with inducible HcRed expression, as described above, and a commercially available EGFP-shRNA fusion construct which targets the cellular *LMNA* gene. The construct was cloned in pRAPtar-2i and -2c. Subsequently, IDRs were constructed which were expected to exhibit inducible HcRed fluorescence in conjunction with inducible or constitutive EGFP expression and parallel *LMNA* knockdown.

**Figure 4:**
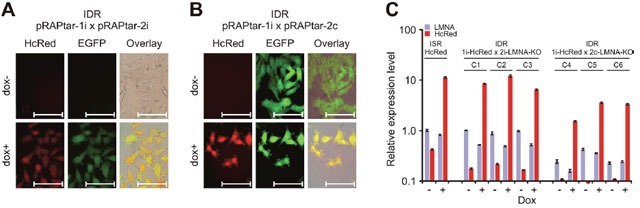
Construction of isogenic double recombinants The ISR with insertion of dox-inducible HcRed was used to generate IDRs via Cre-lox mediated recombination with either **(A)** dox-inducible (pRAPtar-2i) or **(B)** constitutive expression (pRAPtar-2c) of a second sequence. The sequence comprised an emerald GFP (EGFP) reporter gene linked to a cassette for expression of an shRNA targeting the cellular *LMNA* gene. **(A)** Fluorescence microscopy of IDRs with inducible EGFP-shRNA cassette shows induction of both reporters (2 μg/ml dox for maximum induction). Scale bars: 100 μm. **(B)** Fluorescence microscopy of IDRs with constitutive EGFP-shRNA cassette 48 h post induction with 2 μg/ml dox and without induction. Scale bars: 100 μm. **(C)** Quantification of knockdown and overexpression in IDRs. Three clones each (C1-C3 and C4-C6, respectively) were analyzed for knockdown of *LMNA* and induction of HcRed mRNA expression by qRT-PCR. Values are referred to *LMNA* levels in the ISR with HcRed insertion only (without induction). Error bars represent SEM.

Resulting puromycin-resistant IDRs displayed the expected fluorescence patterns (Figure 4A and 4B). qRT-PCR of three individual IDRs for each configuration further suggested uniform performance with downregulation of *LMNA* mRNA to 0.53- to 0.49-fold for the IDRs with inducible EGFP-shRNA expression and 0.43- up to 0.16-fold for IDRs with constitutive EGFP-shRNA expression (Figure 4C). These results indicated that the system could be used efficiently to generate uniform isogenic cell lines with individual or combinatorial molecular traits.

### Construction and screening of an isogenic melanoma cell line library

Our original objective was to develop a system for systematic functional analysis of large gene sets obtained from e.g. genome-wide sequencing studies reporting on recurrent mutations in tumor tissues [1–7]. To establish if our recombination system was able to analyze such large gene sets, we shuttled 108 genes including known tumor suppressors, oncogenes and randomly selected genes (Supplementary Table 2) into the pRAPtar-1i vector and subsequently inserted these into A375-ACLN103 melanoma cells. For 95% of the selected genes point mutations and/or copy number variations in malignant melanoma are reported according to the COSMIC database (http://cancer.sanger.ac.uk/cosmic) [19].

**Figure 5:**
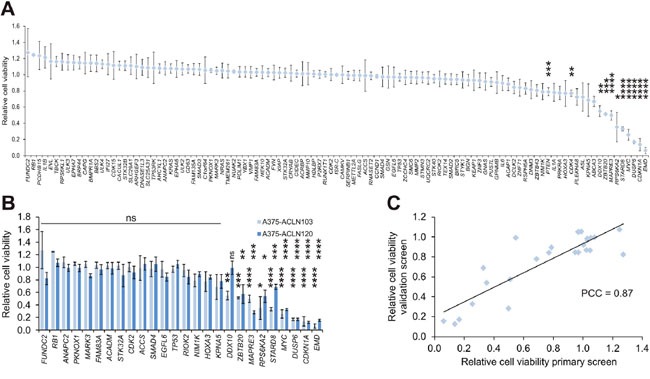
Screen of a melanoma isogenic single recombinant cell library **(A)** A library of 108 A375-ACLN103-derived ISRs was generated and subjected to a cell viability scan as the primary (discovery) screen. Cell viability is displayed relative to ISRs with the empty expression plasmid (pRAPtar-1i) inserted (ISR-C). Eleven primary hits, including known cancer genes like *TP53*, *CDKN1A*, *PTEN* and *MYC*, were identified. **(B)** Validation of a panel of 26 genes in independent ISRs generated with A375-ACLN120 cells. Data from primary screen is included for comparison. **(C)** Correlation plot of validation versus primary screen with Pearson correlation coefficient (PCC) of 0.87. Statistical significance was evaluated using two-tailed Student`s t-tests and is indicated by *: *P* < 0.05; **: *P* < 0.01; ***: *P* < 0.001; ****: *P* < 0.0001. All error bars represent SEM.

Transfection of A375-ACLN103 cells grown in two wells of a 6-well plate per gene was sufficient to yield at least three independent EGFP-negative ISR clones. Three clones per gene were consecutively pooled to minimize any potential random variation. Cells with insertion of the empty pRAPtar-1i vector served as isogenic controls (ISR-C), which allowed normalization for effects introduced by the vector system and/or by changes to the genomic insertion site. Subsequently, we analyzed changes in cell viability before and after induction of gene expression in a discovery screen. We identified 11 genes with significant growth-suppressive effects compared to the isogenic controls (Figure 5A). Many of these effects are in accordance with current literature. For example, A375 cells harbor wild type *TP53*, expressed at substantial levels, but display comparably low *CDKN1A* (p21) protein levels [20]. Accordingly, *TP53* overexpression did not cause significant changes in cell viability, while *CDKN1A* overexpression caused strong growth suppression. MYC has been reported to cause G2 cell cycle arrest in cells with wild type *TP53* [21], which is in accordance with the significant suppression mediated by MYC in the discovery screen. The tumor suppressor *PTEN* exerted a moderate but significant growth suppressive effect (Figure 5A).

We selected a set of 26 genes, including the nine most prominent growth suppressors, for an independent validation by construction and screening of ISRs generated with A375-ACLN120 cells (Supplementary Table 3). Southern blot data indicated a different genomic insertion site as compared to A375-ACLN103 cells (Supplementary Figure 2A and 2B) such that this independent approach was suitable to filter for effects on cell growth caused by the chromosomal location of the insertion site. The validation screen confirmed effects on growth for 25 out of 26 genes. Only *DDX10* was not confirmed, leading to a 96% confirmation rate and a Pearson correlation coefficient of 0.87 (Figure 5B and 5C). In summary, this indicated that ISR libraries can be used efficiently to analyze large gene sets and to identify phenotypic effects in a robust fashion.

### Identification of a potential tumor suppressive network

**Figure 6:**
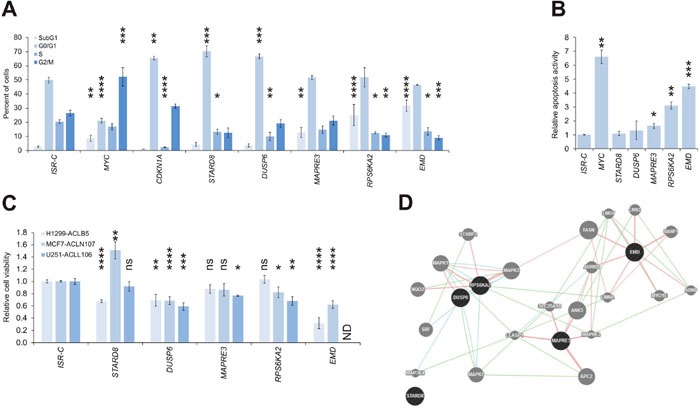
Identification of a tumor suppressive network **(A)** Cell cycle distribution as mean of at least two independent experiments (background: A375-ACLN103). **(B)** Relative apoptosis activity as mean of two independent experiments (background: A375-ACLN103). **(C)** One ACL clone of each of the other three cancer backgrounds was used to create ISRs for the five genes and correspondingly matched negative controls (ISR-Cs). ISRs were subjected to cell viability analyses after induction with dox. The effect of *EMD* in U251-ACLL106 could not be determined (ND) because here no ISR could be obtained. **(D)** Interaction network suggested by GeneMANIA tool (www.genemania.org). Dark gray: query genes; light gray: interacting genes. Genetic, pathway and physical interactions are indicated by green, blue, and red lines, respectively. Statistical significance was evaluated using one-tailed Student`s t-tests for the relative apoptosis activity and otherwise using two-tailed Student`s t-tests and is indicated as *: *P* < 0.05; **: *P* < 0.01; ***: *P* < 0.001; ****: *P* < 0.0001; ns: not significant. All error bars represent SEM.

We selected a subset of the genes with confirmed growth suppressive effects for further analysis. First, we subjected the ISRs generated with A375-ACLN103 to cell cycle analyses. *CDKN1A*, used as positive control, caused the expected G0/G1 arrest. Furthermore, the G2-arrest suggested in the literature for *MYC* overexpression in *TP53* wild type cells was confirmed and accompanied by a significant increase of apoptotic cells (Figure 6A, Supplementary Figure 4). Among the top five growth-suppressing genes tested, *STARD8* and *DUSP6*, a commonly known tumor suppressor [22, 23], arrested cells in G1/G0-phase, while *MAPRE3*, *RPS6KA2* and *EMD* induced apoptosis (Figure 6A, Supplementary Figure 4). Apoptosis-inducing effects were also confirmed in independent assays by quantifying inter-nucleosomal genomic DNA degradation (Figure 6B).

These five genes were further utilized to construct ISRs from ACLs of the other three cancer types, using the inducible vector pRAPtar-1i, to investigate if the observed growth suppressive effects were melanoma-specific. Tumor suppressive effects were confirmed for all five genes in at least one additional cancer type (Figure 6C). ISRs with *EMD* could not be constructed for the brain cancer U251-ACLL106, this was likely due to strong apoptotic effects exerted by low leaky expression levels in the absence of dox. Of note, in MCF7 breast cancer cells, *STARD8* had a growth-promoting effect as opposed to its tumor-suppressive function in melanoma and lung cancer.

GeneMANIA analysis (www.genemania.org) [24] further indicated that *DUSP6*, *EMD*, *MAPRE3* and *RPS6KA2*, but not *STARD8*, are linked in a tumor suppressive network as defined by known genetic, pathway and/or physical interactions (Figure 6D). Taken together, the data confirmed tumor suppressive functions for the five genes, however, *STARD8* may function as context-specific tumor suppressor or oncogene. The unbiased screen identified a potential tumor suppressive network which may be relevant in various cancer types. However, these results have to be confirmed via functional studies.

### The tumor suppressor DUSP6 is a putative synthetic lethal target in melanoma

**Figure 7:**
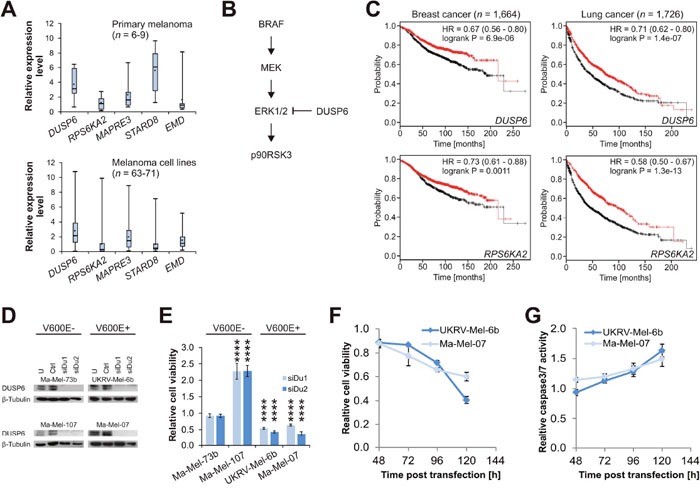
DUSP6 is a context-specific synthetic lethal target in melanoma **(A)** mRNA expression levels for five genes in primary melanoma (top panel) and melanoma cell lines (bottom panel). Case numbers are provided in the diagrams and levels are normalized to the average of two independent samples of NHEM, which were set to 1.0 (not shown). Error bars represent minimum and maximum values. **(B)** Schematic extract from Ras/Raf/MEK/ERK signaling pathway. DUSP6 inhibits p90RSK3 (coded by *RPS6KA2*) activation via ERK1/2 dephosphorylation. **(C)** Kaplan Meier plots for luminal A breast cancer patients (left panels; relapse-free survival) and lung cancer patients (right panels; overall survival). Patients are split according to high (red curve) and low (black curve) expression. HR: hazard ratio with 95% confidence interval. **(D)** Western blot analyses of siRNA-mediated DUSP6 knockdown in four selected melanoma cell lines with confirmed *BRAF* mutation status [43]. U: untreated; Ctrl: non-targeting control siRNA; siDu1 and siDu2: two DUSP6-targeting siRNAs. **(E)** Relative cell viability after DUSP6 knockdown referred to Ctrl. **(F)** Time course of relative cell viability in the two susceptible cell lines, and **(G)** corresponding Caspase-3/7 activity assessed in parallel and normalized to cell viability. Statistical significance was evaluated using two-tailed Student`s t-tests and is indicated by *: *P* < 0.05; **: *P* < 0.01; ***: *P* < 0.001; ****: *P* < 0.0001. All error bars represent SEM unless otherwise indicated.

Analyses in primary melanoma and melanoma cell lines indicated that *MAPRE3* and *EMD* mRNA levels were not substantially changed compared to primary normal human epidermal melanocytes (NHEM). While *STARD8* levels were upregulated in primary tumors and decreased in cell lines (Figure 7A), *RPS6KA2* levels were normal or decreased, however, *DUSP6* mRNA expression was consistently elevated (Figure 7A).

Activating BRAF mutations, such as the common V600E mutation, result in increased MEK activation, which leads to ERK1/2 phosphorylation. Phospho-ERK1/2 in turn activates downstream targets via phosphorylation, among these p90RSK3 which is coded by *RPS6KA2*. In this regulatory circuit, DUSP6 functions as tumor suppressor in limiting ERK1/2 activity via dephosphorylation (Figure 7B) [23, 25, 26]. In accordance with its role as an ERK1/2 downstream target, *RPS6KA2* has been associated with oncogenic functions by various groups [27–29]. Here, our results indicated that increased p90RSK3 activity triggers apoptosis (Figure 6A and 6B), suggesting that p90RSK3 may also function as a tumor suppressor in specific scenarios. Survival analyses available for breast and lung cancer patients [30] supported this hypothesis as high expression of both *DUSP6* and *RPS6KA2* were associated with significantly improved survival in both cancer types (Figure 7C).

We therefore hypothesize that elevated DUSP6 levels in melanoma are one mechanism for compensating BRAF V600E hyperactivation, which might otherwise trigger apoptosis via ERK1/2 downstream targets. To investigate this, we performed an siRNA-mediated knockdown of DUSP6 in BRAF wild type melanoma. As expected for a tumor suppressor, the knockdown either promoted cell growth or did not exert any effect, while the same knockdown in BRAF V600E melanoma with high DUSP6 expression caused cell death via induction of apoptosis (Figure 7D-7G). This indicated inactivation of tumor suppressors, specifically DUSP6, as a counter-intuitive, but conceivable, synthetic lethal therapeutic concept in a subset of melanomas.

## DISCUSSION

Our present study was initiated to investigate how large gene sets, resulting from genome-wide cancer mutation screens, can be functionally analyzed with a high degree of efficiency and standardization. There is a continuously growing demand for such functional genomic approaches because large numbers of driver genes have been classified solely based on statistical analysis and bioinformatic filtering processes [1–7]. However, the vast majority of the identified genes and their mutations have unknown functions in general and in cancer in particular. Finally, as discussed below, the identification of oncogenes or tumor suppressors in one cancer (sub)type does not necessarily translate to other cancer (sub)types.

Several strategies for systematic functional analysis are based on currently available techniques. Among these are genome-editing techniques that allow for the introduction of genetic changes to obtain isogenic cells. While these techniques provide site-specific integration they may require the screening of hundreds of cell clones to identify corresponding cells carrying the desired knockdown or specific mutation [10–14]. We propose the construction of accepter cell lines (ACLs), which greatly simplifies the subsequent insertion of one or several genes of interest. We demonstrate that the first step to creating suitable ACLs experiences the same low efficacy as genome-editing. Even with an improved vector system, it required scanning of 374 clones to derive 8 suitable ACLs, corresponding to an efficacy of 2%. However, the consecutive insertion of target genes into ACLs demonstrated a success rate of nearly 100%.

Within the >100 constructed ISRs, only an isogenic U251 cell line for induced overexpression of *EMD* could not be generated, conceivably due to the strong apoptotic effects caused by the gene. Thus, the proposed strategy enables the efficient generation of permanent isogenic cell line libraries, which can successively be interrogated into various functional assays to analyze effects on e.g. proliferation, invasion/migration, apoptosis, or tumorsphere formation. We analyzed effects on cell viability as a typical readout and achieved a 96% concordance rate between the discovery screen and the validation screen in an independent ACL derived from A375 melanoma cells.

As proof of concept, we constructed an isogenic melanoma cell line panel for a set of 108 genes, which were subsequently analyzed in a cell viability screen. We acknowledge that cell viability encompasses only one functional aspect among many that contribute to tumorigenesis. However, once established, the cell line libraries can be extensively studied using various additional functional assays.

Our results further demonstrated that five genes selected for their strong effect on cell viability also exerted effects in other cancer types. One of the five genes was *STARD8* (also known as *DLC3*), which functions as a Rho-specific GTPase-activating protein involved in endocytic trafficking [31]. Its inactivation may prevent EGFR degradation and interfere with adherens junction integrity, in particular with E-cadherin function [31, 32]. One study reported a tumor suppressive role of *STARD8*, which is in accord with our results in A375 cells [33]. However, Durkin *et al*. observed indications for a tumor suppressive role of *STARD8* in MCF7 cells [33], whereas our results suggested a growth-promoting effect. The contrasting data could be a result of Durkin *et al*. using the longer DLC3α transcript variant [33], while we used the alternatively spliced shorter DLC3β variant. *MAPRE3* (also known as *EB3* or *EBF3*) codes for a microtubule end-binding protein that stabilizes focal adhesions and has been demonstrated to trigger apoptosis in cancer types other than those analyzed here [34–36]. *EMD* codes for emerin, which is affected by mutations in X-linked Emery-Dreifuss muscular dystrophy. While EMD-deficient fibroblasts were recently shown to proliferate abnormally [37], the gene has not yet been linked to cancer. No consistent pattern of changes in mRNA expression levels were observed for these three genes in melanoma, such that their putative role in this cancer type remains elusive.

We further analyzed *DUSP6*, which is a well-described tumor suppressor engaged in a regulatory loop with ERK1/2. DUSP6 counteracts Ras/Raf/MEK/ERK signaling by ERK1/2 desphosphorylation [23, 26]. In our study, it exerted tumor suppressive functions in all four cancer types analyzed. Notably, we uncovered an unexpected opportunistic oncogenic role for DUSP6 that may involve downstream signaling via p90RSK3, which is activated by ERK1/2-mediated phosphorylation. Inactivation of p90RSK3 acts synergistically with EGFR-inhibition, thus posing a potential synthetic lethal drug target for overcoming PI3K-inhibitor resistance in breast cancer [27, 28]. Of note, *RPS6KA2* was also determined to be a downstream target of BMI1 in glioblastoma stem cells [29]. These findings indicate a role as an oncogene. In contrast, Bignone *et al*. reported that *RPS6KA2* is a tumor suppressor that triggers G1-arrest and apoptosis in ovarian cancer [38].

We also observed *RPS6KA2*-mediated apoptosis in our studies, which led us to investigate the knockdown effect of the upstream tumor suppressor DUSP6, which we expected to result in RPS6KA2 hyperactivation via ERK1/2. Our hypothesis led to the identification of DUSP6 as a potential synthetic lethal target in melanoma with BRAF V600E mutation and high expression of DUSP6, suggesting that, in certain scenarios, this tumor suppressor may serve as drug target. This counter-intuitive proposition might be supported by recent findings that indicate less strict delineations between tumor suppressors and oncogenes. The concept of *TP53* gain-of-function mutants has been revitalized and the functions of oncogenic TP53 are currently under intensive investigation [39–41]. Importantly, members of the DUSP protein family, including DUSP6, have recently been proposed as therapeutic targets for glioblastoma multiforme, where DUSP6 causes tumor-promoting effects and chemoresistance [42, 43].

A distinct advantage to the presented strategy is its support for studying combinatorial traits, e.g. by the overexpression of one target gene and the parallel knockdown of a second target gene. We demonstrated this by constructing IDRs with fluorescence reporter expression and a parallel *LMNA* knockdown via subsequent recombination of both target sequences into the ACL genome. Thus, the system can, in principle, be used for large combinatorial studies such as a systematic screen for synthetic lethal targets in cancer.

In conclusion, we demonstrate a robust and flexible strategy for the construction of isogenic cell line libraries and establish its applicability for systematically screening larger gene sets commonly recovered from genome-wide sequencing studies. The results of a systematic proof-of-concept cell viability screen led to subsequent hypothesis-based experiments, which identified *DUSP6* as context-specific synthetic lethal target in melanomas with BRAF V600E mediated ERK1/2 activation.

## MATERIALS AND METHODS

### Cell lines and patient samples

Cell lines from Table 1 were obtained and cultivated according to the instructions from ATCC. Primary melanoma samples and patient-derived melanoma cell lines are part of the panel previously described [44]. All tumor samples and clinical data were collected with Institutional Review Board approval and patient's informed consent.

### Transfection of acceptor plasmids

For comparison of pRAPacc1/2 to the pFRT/lacZeo, 2 × 10^6^ cells were transfected with 4 μg plasmid DNA in 100 μl Nucleofector Solution (Lonza) via nucleofection, applying the conditions recommended by the supplier. Transfected cells were seeded into two wells of a 6-well plate per cell line and 24 h later zeocin or G418 selection was introduced at concentrations previously determined in titration experiments. After a further 48 h, cells were transferred to 10 cm cell culture dishes and cultivated for 2 weeks for subsequent counting of reporter-positive clones. Cells transfected with pFRT/lacZeo were evaluated by conventional β-Gal staining, while cells transfected with pRAPacc1/2, were scored according to the number of green fluorescent colonies observed by fluorescence microscopy. Experiments were performed 2-3 times to calculate average values.

### Generation of isogenic recombinant cell lines

Genes were shuttled into pRAPtar-1/2 vectors via the GATEWAY system (Invitrogen) according to the instructions of the supplier. The respective expression plasmid DNA was mixed at 1:5 ratio with either pOG44 for Flp-mediated recombination or pGK-Cre for Cre-mediated recombination. Consecutively, 1 × 10^6^ cells were seeded in 6-well plates and transfected with 5 μg plasmid mixture using Lipofectamine according to manufacturer's instructions. Three days post transfection, the selection antibiotic was added (hygromycin for ISRs and puromycin for IDRs) according to individual optima determined for the ACLs. In the majority of the transfections at least three non-green fluorescent clones per 2 wells emerged, which were isolated and propagated separately until pooled for further analyses. For gene and cell line resources management, we utilized the OpenLabFramework software [45].

### Southern blot analyses

Southern blot analyses were performed to identify ACLs with single integration and to confirm single insertion into the authentic locus for selected recombinants. For single integration 10 μg genomic DNA was digested with *Pst*I and for single insertion 15 μg genomic DNA was digested with *Kpn*I, followed by gel electrophoresis on 1.2 % agarose gels and conventional Southern blotting. Standard random hexamer-based radioactive labeling was applied to generate an *EGFP*-specific probe for analyses of ACLs and a probe targeting the hygromycin resistance gene for analyses of *TP53*-ISRs.

### Knockdown constructs

We utilized the cassette of the BLOCK-iT™ Pol II miR-*LMNA* Validated miRNA Control Vector (Thermo Fisher Scientific), where the *LMNA*-targeting shRNA is cloned behind emerald GFP (EGFP). The insert was shuttled into pRAPtar-2i and -2c vectors, which consecutively were co-transfected with pGK-Cre plasmid into A375-ACLN103 ISR cells already carrying the inducible HcRed reporter in the first site. The HcRed ISR cells served as reference point for comparison in these analyses. Because use of pRAPtar-2i inserts a second Tet-repressor and the two CMV promoters on pRAP-tar-1 and pRAPtar-2 might compete for each other, a higher dox concentration, arbitrarily set to 2 μg/ml, was used for these experiments.

### Fluorescence microscopy

Images of fluorescent cell lines were taken at 60 x magnification (scale bars provided in figures) with an Olympus IX71 microscope, using the filter sets U-MNIBA3 (green fluorescence) and U-MWU2 (red fluorescence) and the Cell soft pro software (Olympus) at uniform exposure times 500 ms for green and 1000 ms for red fluorescence. Image acquisition of IDRs in Figure 3B was performed by using the automated contrast enhancement of the software due to weaker HcRed fluorescence in IDRs.

### Quantitative reverse transcription polymerase chain reaction

For quantitative reverse transcription polymerase chain reaction (qRT-PCR) total RNA was purified using TRIzol reagent (Invitrogen), DNAse treated, and subjected to oligo-dT-primed reverse transcription according to standard protocols. Analyses were performed in triplicate wells with 10 ng cDNA, using Human *ACTB* and/or *GAPDH* Endogenous Control assays (Applied Biosystems) as references for normalization. The PCR was performed using a 7500 Real-Time PCR System (Applied Biosystems) under the following conditions: 60°C for 15 min (1 cycle) and 95°C for 15 sec, 60°C for 1 min (40 cycles). Data was analyzed using qbasePLUS evaluation software (Biogazelle). The following gene-specific assays (Applied Biosystems) were used: Hs99999147_m1 for *TP53*, Hs00355782_m1 for *CDKN1A*, Hs00153462_m1 for *LMNA*, Hs00169257_m1 for *DUSP6*, and a custom-made assay was used for HcRed mRNA detection (forward primer: 5′-GGAGAGAACCACCACCTACGA-3′; reverse primer: 5′-CCTCCAGGCTGGTGTCC-3′; labeled probe: FAM-5′-ACGGCGGCATCCTGA-3′–NFQ).

### Flow cytometry

For flow cytometric analyses, cells were trypsinized to a single cell suspension, centrifuged (270 g for 5 min), resuspended in Hanks Balanced Salt Solution (HBSS) and stored on ice until analyzed with a FACSCalibur flow cytometer (Becton Dickinson). Cell debris and dead cells were excluded from the analyses via forward and side scatter parameters. EGFP was detected at 488 nm in the FL1 channel, while HcRed was detected at 640 nm in the FL4 channel. Per analysis 10,000 cells were evaluated. HcRed levels were analyzed after 48 h with induction by various dox-concentrations.

For cell cycle analyses, we incubated the cells for 4 days in the presence of 50 ng/ml dox. The cells (about 5 × 10^5^ cells) were then trypsinized, gently pelleted by centrifugation, and fixed with methanol for 6 h at -20°C. After removal of methanol, cells were resuspended in 7-aminoactinomycin D (7AAD) solution (5 μg/ml 7AAD, 1 mg/ml RNase A in PBS) and incubated for 30 min at 37°C followed by 90 min at 4°C. Fluorescence was evaluated as described above (detection at 650 nm; FL3 channel).

### Cell viability assays

We seeded 1,000 cells per well in 96-well plates and determined cell viability after 5 d incubation by addition of 20 μl CellTiter-Blue reagent (Promega) per well. After 3 h incubation, fluorescence was measured at 550/610 nm in a Fusion Fluorometer (Packard Biosciences). Wells containing medium but no cells and processed in parallel were used for blank subtraction. To determine relative cell viability robustly, we first calculated relative viability of ISR cells without dox addition and with addition of 50 ng/ml dox relative to two independent empty vector control ISRs (ISR-C) processed in parallel. We then calculated the ratio of dox+/dox- cell viability, which should normalize for all factors contributed by vector components and/or inherent random variation among ISRs. Experiments were performed at least three times in triplicate. In the primary screen, only ISRs, which displayed a 20% or more change in cell viability were tested for statistical significance against isogenic controls.

### Apoptosis assays

Relative apoptosis activity was determined using the Cell Death Detection ELISAPlus Kit (Roche). In brief, 10^4^ cells per well were seeded in 12-well plates and cells were induced with 50 ng/ml dox 24 h post seeding. Determination of apoptosis activity followed 48 h post induction according to the instructions of the supplier. After subtraction of blank (medium-only) values, relative apoptosis activity was calculated normalized to an isogenic negative control cell line with the empty vector construct inserted (ISR-C).

Caspase-3/7 activity after DUSP6 knockdown was determined using the Apo-ONE Homogeneous Caspase-3/7 assay according to the protocol of the provider (Promega) and the fluorescence signal was detected at the wavelengths 485/535 nm with a Victor3 multi-label counter (PerkinElmer). The Caspase signal was normalized to the relative cell number analyzed in parallel using the fluorometric CellTiter-Blue reagent (Promega) as described above and finally referred to the values obtained for the non-targeting negative siRNA control (see below).

### DUSP6 knockdown

For DUSP6 knockdown, cells were transfected with 25 nM of siDu1 (sense: 5`-GUGCAACAGACUCGGAUGGUAtt-3`; anti-sense: 5`-UACCAUCCGAGUCUGUUGCACtt-3`), siDu2 (sense: 5`-AGCUCAAUCUGUCGAUGAAtt-3`; anti-sense: 5`-UUCAUCGACAGAUUGAGCUtc-3`), or the non-targeting AllStars negative control siRNA (Qiagen), respectively, using the Lipofectamine RNAiMAX transfection reagent (Invitrogen) according to manufacturer's instructions. Transfection experiments were performed at least three times in triplicate in 24-well format using 12,500 cells per well. The medium was replaced 24 h post transfection and cells were cultured for an additional 48 h for protein extraction or another 96 h for cell viability and apoptosis assays.

### Western blot analyses

To confirm the knockdown of DUSP6, total protein was harvested with RIPA buffer 48 h post siRNA transfection. Proteins (20 μg per sample) were separated by SDS-PAGE via an 8 % Precise protein gel (Life Technologies) and blotted onto a Immobilon-P transfer membrane (Millipore). Detection of DUSP6 was performed using mouse monoclonal antibody (1:1000; Abcam ab54940) and HRP-conjugated rabbit anti-mouse IgG (H+L) secondary antibody (1:5000; Jackson Immunoresearch 315-035-003), and finally visualized with SuperSignal West Dura Extended Duration Substrate (Life Technologies). The antibody was afterwards removed from the membrane with 10 M urea, 45 mM SDS followed by washing steps with 50 % ethanol, 10 % acetic acid. Consecutively, the membrane was re-probed with a mouse monoclonal anti-beta tubulin antibody (1:1000; Abcam ab7792) and the same secondary antibody as before to confirm equal loading amounts of protein lysate.

## SUPPLEMENTARY DATA FIGURES AND TABLES





## Abbreviations

ACL: acceptor cell line
Cas9: CRISPR-associated protein-9 nuclease
CRISPR: Clustered regularly-interspaced short palindromic repeats
Flp: flippase
FRT: flippase recognition target
IDR: isogenic double recombinant cell
ISR: isogenic single recombinant cell
ISR-C: isogenic single recombinant cell negative control
NGS: next-generation sequencing
NHEM: primary normal human epidermal melanocytes
shRNA: short hairpin RNA
siRNA: short interfering RNA
TALEN: Transcription Activator-Like Effector Nuclease
