# Supplementary material for: Systematic screening of isogenic cancer cells identifies DUSP6 as context-specific synthetic lethal target in melanoma
Source: Oncotarget. 2017 Mar 2;8(14):23760–74. doi: 10.18632/oncotarget.15863 (PMC5410342; doi:10.18632/oncotarget.15863)
Supplement: Supplementary file 1 [file oncotarget-08-23760-s001.pdf]

Systematic screening of isogenic cancer cells identifies DUSP6 as context-specific synthetic lethal target in melanoma

SUPPLEMENTARY DATA

REFERENCES

1. Senecoff JF, Bruckner RC, Cox MM. The FLP recombinase of the yeast 2-micron plasmid: characterization of its recombination site. Proc. Natl. Acad. Sci. USA. 1985; 82:7270-7274.
2. Zhang Z, Lutz B. Cre recombinase-mediated inversion using lox66 and lox71: method to introduce conditional point mutations into the CREB-binding protein. Nucleic Acids Res. 2002; 30:e90.
3. Kwaks TH, Barnett P, Hemrika W, Siersma T, Sewalt RG, Satijn DP, Brons JF, van Blokland R, Kwakman P, Kruckeberg AL, Kelder A, Otte AP. Identification of anti-repressor elements that confer high and stable protein production in mammalian cells. Nat. Biotechnol. 2003; 21:553-558.
4. Kuhstoss S, Rao RN. Analysis of the integration function of the streptomycete bacteriophage phi C31. J. Mol. Biol. 1991; 222:897-908.

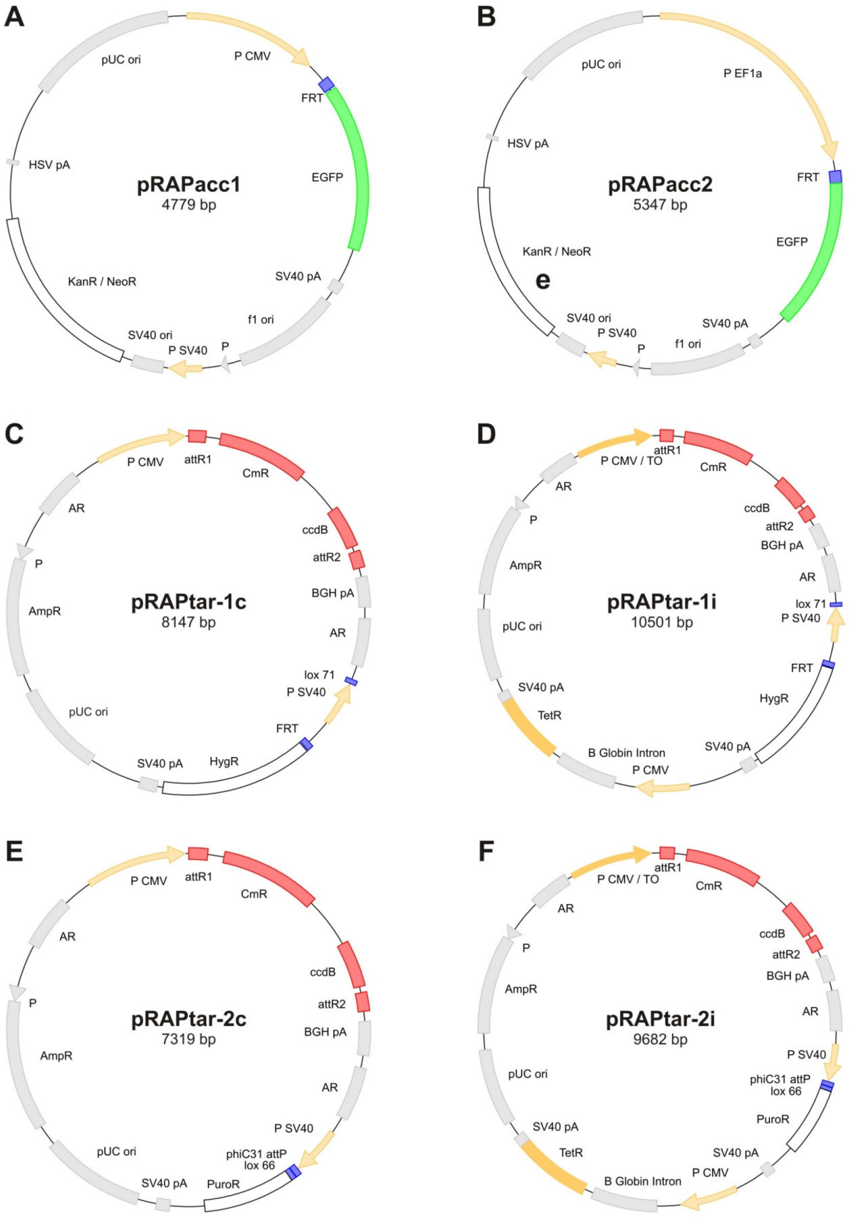

Supplementary Figure 1: Schematic plasmid maps. For abbreviations of genetic elements refer to Supplementary Table 1. **(A)** pRAPacc1 and **(B)** pRAPacc2 used for stable insertion to generate acceptor cell lines and providing a FRT-sequence[1] for consecutive site-specific recombination. **(C)** pRAPtar-1c and **(D)** pRAPtar-1i used for construction of isogenic single recombinants with constitutive and dox-inducible overexpression, respectively. The FRT-site is used for Flp-recombinase mediated recombination into the pRAPacc1 or pRAPacc2 vector inserted into the host cell`s genome. Correct recombination puts the promoter/start codon-deficient hygromycin resistance gene (Hyg^R) under the control of the promoter/start codon that was previously driving EGFP expression. Simultaneously, a lox71-sequence[2] for Cre-mediated recombination of a second plasmid is provided, which is linked to the simian virus 40 promoter (P SV40). AttR1 and attR2 flank a GATEWAY system-compatible cassette that would be exchanged for the sequence of interest via *in vitro* recombination. Anti-repressor elements (AR) are included to suppress promoter silencing after insertion into the genome[3]. Finally, pRAPtar-1i carries a Tet-repressor (TetR) in *cis*, so that the same acceptor cell line can be used to derive isogenic single recombinants with either constitutive or inducible expression in a single step. **(E)** pRAPtar-2c and **(F)** pRAPtar-2i for construction of isogenic double recombinant cells (IDRs) with constitutive or inducible expression, respectively, of a second sequence. The lox66-site is cloned in front of a puromycin resistance gene ($Puro^R$), which, after Cre-mediated recombination, is placed under the control of the P SV40 of the pRAPtar-1 plasmid. A phiC31 attP site[4] linked to another P SV40 would then be available for insertion of a third plasmid via site-specific recombination.

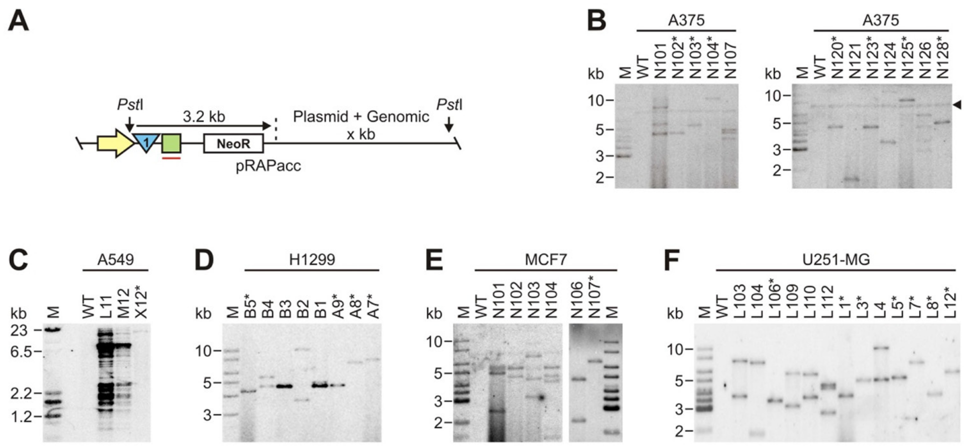

Supplementary Figure 2: Identification of acceptor cell line clones with single integration. (A) Intended configuration of pRAPacc plasmid in the genomic context. The restriction enzyme *Pst*I cuts only once in the pRAPacc plasmid, i.e. in front of the FRT-site. If FRT-site, EGFP and the neomycin resistance gene are inserted intact, a restriction fragment of 3.2 kb from the vector (indicated by a dashed line) plus the size defined by the next *Pst*I recognition site in the host cells` genome should provide a result that is recognized by the radioactively labeled EGFP probe (indicated as red line). Thus, only clones displaying a single hybridizing band larger than 3.2 kb were considered to have the intended single integration event. Yellow arrow: CMV promoter; blue triangle: FRT-site; green square: EGFP open reading frame; white rectangle: neomycin resistance gene. **(B-F)** Southern blot analyses including relevant clones with single integration of pRAPacc plasmids. Clones with single integration are marked with asterisks. Note that the size marker cross-hybridizes to variable extent with the EGFP probe. Images were cropped, size- and brightness-adjusted. Brightness was adjusted uniformly over the entire image for each of the images. The arrowhead in (B) depicts an unspecific band in the genomic DNA of A375 cells, cross-reacting with the EGFP probe. The band was observed throughout all clones and in the original unmodified A375 cell line (WT). Note that two of the three clones shown for A549 cells have massive multiple insertions as indicated by various bands of different size and increased band intensities. By contrast, the vast majority of the other cell lines displayed 2-3 bands pointing to more than one integration event. Clones with putative multiple insertions were excluded from further analyses because here the action of Flp-recombinase could produce uncontrollable chromosomal rearrangements. WT: corresponding unmodified cell line as negative control; M: size marker.

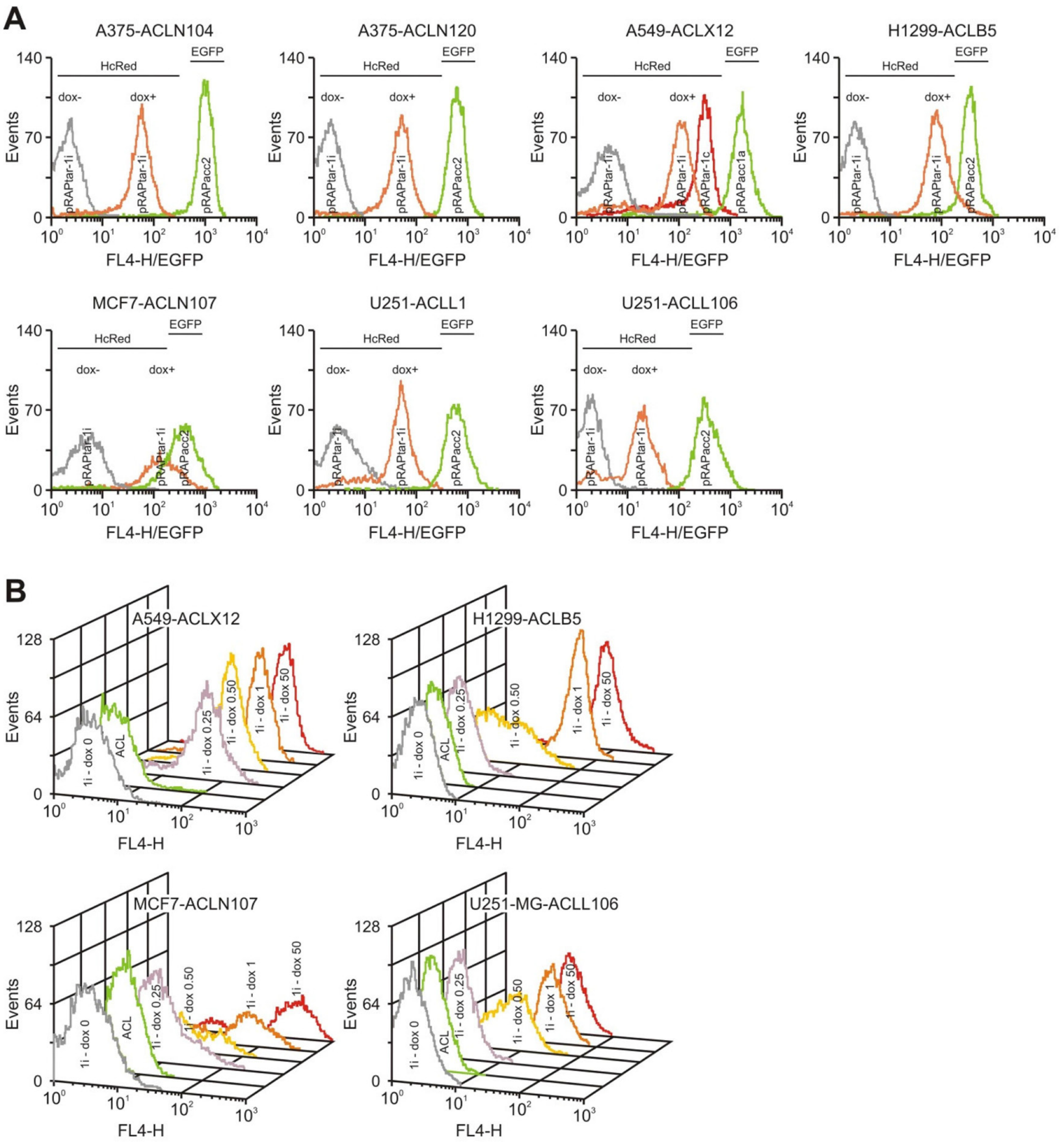

Supplementary Figure 3: Flow cytometric analyses of reporter gene expression in acceptor cell lines. (A) Overlay of profiles for EGFP (acceptor cell lines; ACLs) and HcRed expression as determined by flow cytometry. EGFP expression (green curve) emerges from the inserted pRAPacc1/2 plasmid prior to site-specific recombination. After successful recombination EGFP expression is eliminated. Inducible expression in isogenic single recombinants (ISRs) was evaluated with the HcRed reporter gene shuttled into the pRAPtar-1i vector in the absence of doxycycline (dox-) and 48 hours after induction with 50 ng/ml doxycycline (dox+). For A549-ACLX12 the constitutive pRAPtar-1c vector with HcRed was additionally evaluated in flow cytometry. **(B)** Quantitatively tunable induction of gene expression was analyzed and confirmed for one ACL clone per cancer cell line using HcRed in pRAPtar-1i and applying the different doxycycline concentrations depicted in ng/ml at the respective profile. The green curve marked as ACL is HcRed fluorescence of the acceptor cell line prior to recombination, serving as negative control.

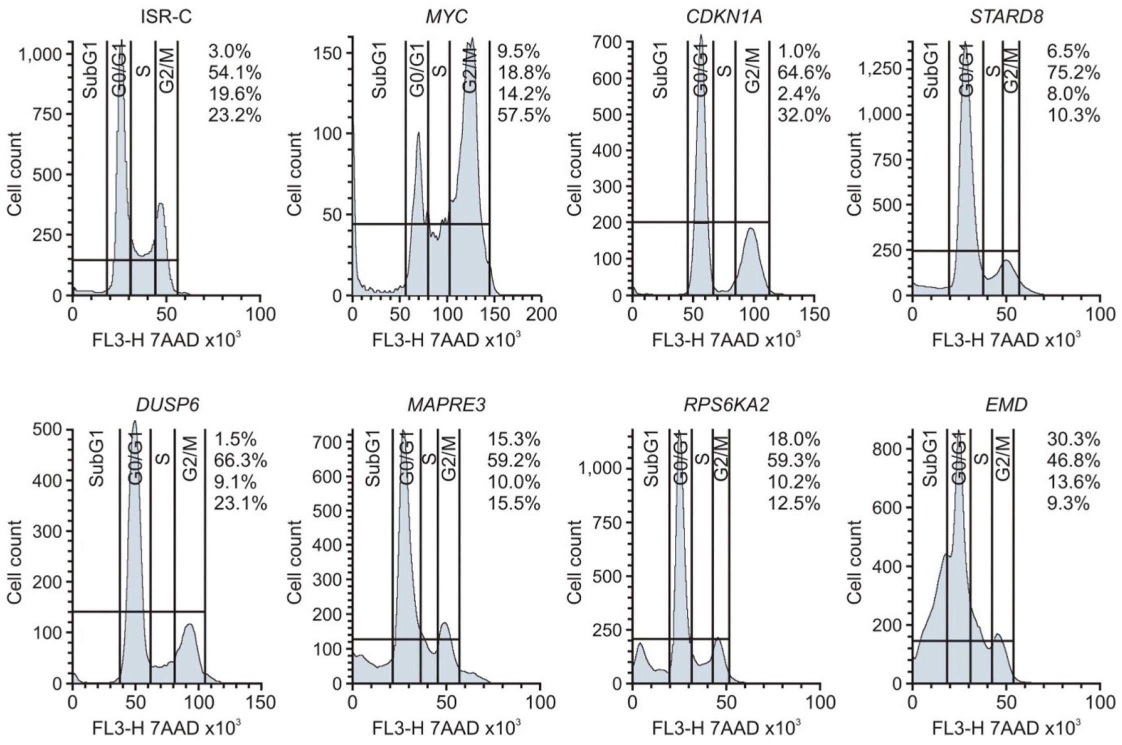

Supplementary Figure 4: Cell cycle analyses. Representative flow cytometry profiles after 7AAD staining of ISRs from A375-ACLN103. Percentages of cells in the respective phase are depicted at the right (from top to bottom: SubG1, G0/G1, S, G2/M phase).

Supplementary Table 1: Abbreviations for genetic elements in the plasmid maps

Element	Function
AR	Anti-repressor element
Att	Attachment sites of the GATEWAY™ system
AmpR	Ampicillin resistance gene
BGH pA	Bovine growth hormone polyadenylation signal
B Globin Intron	Rabbit B Globin Intron
ccdB	Lethal gene targeting mutant DNA gyrase of *E.coli* DB3.1
CmR	Chloramphenicol resistance gene
EGFP	Enhanced green fluorescent protein gene
f1 ori	f1 single strand DNA origin of replication
FRT	Flippase Recognition Target (FRT) site
HSV pA	Herpes simplex virus polyadenylation site
HygR	Hygromycin B resistance gene
KanR	Kanamycin resistance gene
Lox 66	Recognition sequence Lox 66 for Cre-mediated recombination
Lox 71	Recognition sequence Lox 71 for Cre-mediated recombination
NeoR	Neomycin resistance gene
P	Bacterial promoter
P CMV	Cytomegalovirus promoter
p CMV/TO	CMV promoter containing Tet-operator sequences
p EF1a	Human Elongation Factor 1-alpha promoter
p SV40	Simian virus 40 promoter
phiC31 attP	Phage phiC31 integrase recognition sequence
pUC ori	pUC origin of replication
PuroR	Puromycin resistance gene
SV40 pA	Simian virus 40 polyadenylation signal
TetR	Tet-repressor

Supplementary Table 2: Gene Set and Discovery Screen

See Supplementary File 1

Supplementary Table 3: Gene Set Validation Screen

See Supplementary File 1
